# 533. Protocol for and Efficacy of Monoclonal Antibody (mAb) Treatment of SARS-CoV-2 at a VA Medical Center

**DOI:** 10.1093/ofid/ofab466.732

**Published:** 2021-12-04

**Authors:** Phuong Khanh Nguyen, Thomas D Dieringer, Adela Greeley, Suny Kun, Feliza Calub, My-Phuong Pham, Christopher J Graber, Matthew B Goetz, Matthew B Goetz, Kevin Ikuta

**Affiliations:** 1 VA Greater Los Angeles Healthcare System, Los Angeles, California; 2 UCLA, Mountain View, California; 3 Greater Los Angeles VA, Los Angeles, California; 4 VA Greater Los Angeles Healthcare System/UCLA, Los Angeles, California; 5 VA Greater Los Angeles Healthcare System and David Geffen School of Medicine at UCLA, VA-CDC Practice-Based Research Network, Los Angeles, California

## Abstract

**Background:**

Bamlanivimab and casirivimab/imdevimab were the first monoclonal antibodies (mAb) developed against SARS-CoV-2 and proved beneficial early in the course of infection. However, real-world administration of these therapies presents logistical challenges. We present our experience implementing mAb treatment at a large VA Medical Center and review the efficacy of therapy in preventing hospitalization from COVID-19 in a closed healthcare system.

**Methods:**

All positive outpatient COVID tests performed at VA Greater Los Angeles Healthcare System (GLA) were reviewed by the Emergency Medicine (EM) and Infectious Diseases (ID) Sections for mAb eligibility beginning 12/2/2020. Due to limited supply, treatment was prioritized for patients at highest risk of developing severe disease, as determined by EM/ID with input from a machine learning ensemble risk estimation model produced by VA National Artificial Intelligence Institute (Figure 1). If a patient declined or did not reply, treatment was offered to the next patient on a ranked eligibility list. Those who declined or were eligible but not treated were included in the analysis. Patients were excluded if they were hospitalized before treatment was offered. We collected data on age, comorbidities, date of diagnosis, and admission at 30 days after diagnosis. A multivariate log binomial regression was performed to determine the relative risk of admission within 30 days of diagnosis for those who received mAb therapy as compared to those who did not, adjusting for age and comorbidity. All analysis was done in R (version 4.0.5).

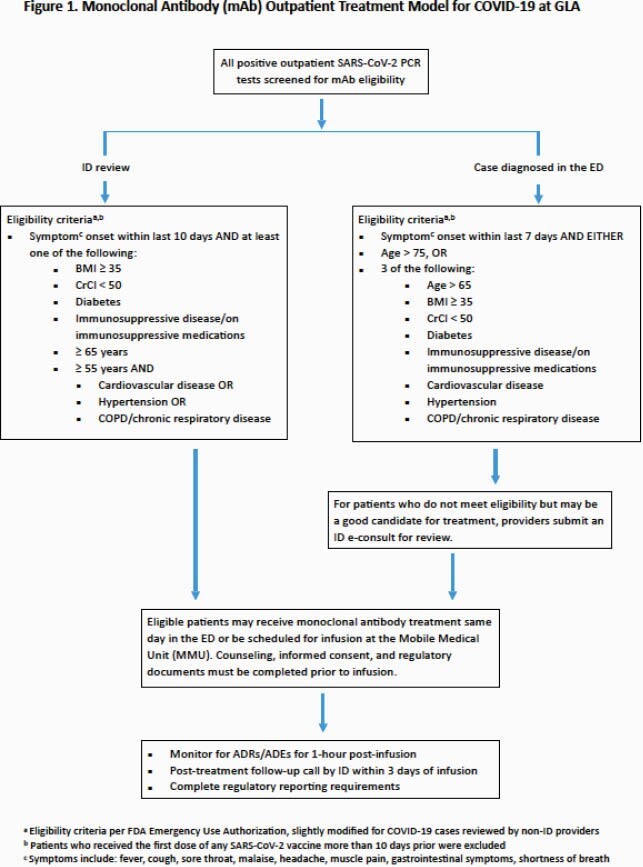

**Results:**

139 patients met inclusion criteria. 45 (32%) received mAb therapy, 48 (35%) declined mAb therapy, and the remaining 46 (33%) either did not respond or were not offered mAb therapy. Hospitalizations following diagnosis in each group are illustrated in Figure 2. There was a trend towards reduced absolute and relative risk of hospitalization (Table 1). There were no anaphylactic events in patients who received mAb therapy.

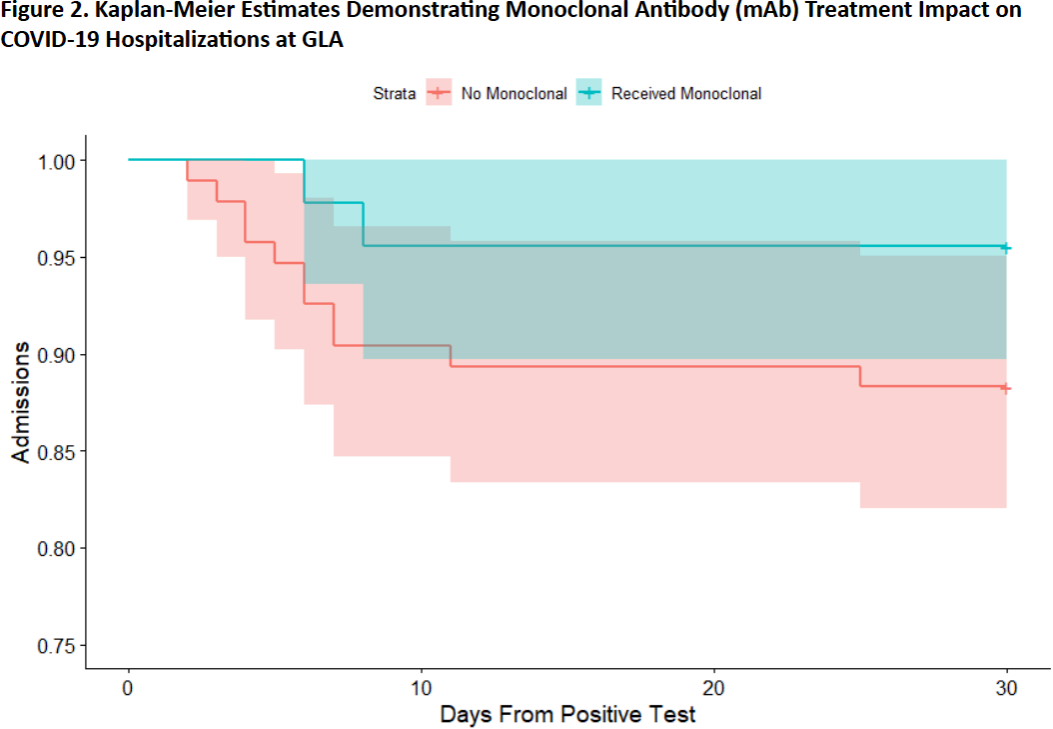

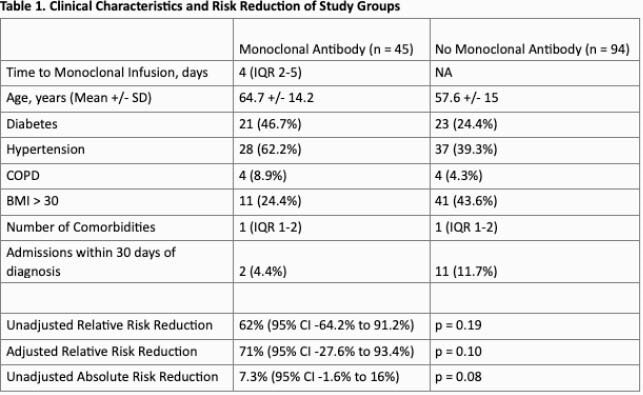

**Conclusion:**

At our facility, a system for rapid identification of candidates and a coordinated distribution plan was essential in ensuring timely administration of mAb therapy to eligible patients. Administration of mAb showed a trend towards decreased risk of hospitalization due to SARS-CoV-2.

**Disclosures:**

**Adela Greeley, MD**, **Kite** (Other Financial or Material Support, My spouse is an employee) **Matthew B. Goetz, MD**, Nothing to disclose

